# Decision‐Making Competence in Health Behavior Among Rural Older Adults with Cognitive Decline

**DOI:** 10.1002/alz.088579

**Published:** 2025-01-09

**Authors:** Myonghwa Park, Heejung Kim, Jinju Kim, Eunkyung Seo, Wonmi An

**Affiliations:** ^1^ Chungnam National University, College of Nursing, Daejeon Korea, Republic of (South)

## Abstract

**Background:**

The aging population in rural areas is a unique health challenge. Older adults with cognitive decline are particularly vulnerable due to limited access to medical resources and health‐related information, a disparity more pronounced in rural compared to urban settings. This study investigates key factors influencing perceived decision‐making competence for health behavior in this demographic.

**Method:**

This study was conducted as a secondary analysis of data from a larger health promotion project in a rural area of South Korea. The data of 170 older adults, aged 60 and above was analyzed. These individuals were identified as having suspected cognitive impairments based on the 10‐item cognitive function screening test. The study focused on assessing various factors such as cognitive function, social support, quality of life, and perceived decision‐making competence for health behaviors.

**Result:**

The assessment of decision‐making competence for health behavior was conducted using a scale ranging from 1 to 5, with the mean score being 3.03 ± 0.66. Detailed analysis showed varying levels of competence across different health behaviors: regular exercise (2.54±1.13), healthy diet (2.92±1.05), brushing teeth (2.87±1.19), regular health checkup (3.05±1.09), infection prevention (3.48±1.10), social activity (3.45±1.11), and health education participation (2.91±1.01). This revealed a pattern where participants showed lower competence in areas requiring more active engagement, like regular exercise, and higher competence in more passive or socially facilitated behaviors like infection prevention and social activity. Furthermore, a positive correlation was observed between the levels of social support, the quality of life (as measured by the EQ‐5D), and the decision‐making competence for health behaviors.

**Conclusion:**

The study’s findings highlight significant variations in decision‐making competence among rural older adults with cognitive decline, influenced by the type of health behavior and the level of social support and quality of life. These insights emphasize the need for tailored health promotion strategies that consider these variables. Future research should aim to deepen the understanding of these relationships, particularly by comparing this group with older adults without cognitive impairments or in urban area. and exploring the causal factors behind different levels of decision‐making competence.

